# Left Ventricular Trabeculations Decrease the Wall Shear Stress and Increase the Intra-Ventricular Pressure Drop in CFD Simulations

**DOI:** 10.3389/fphys.2018.00458

**Published:** 2018-04-30

**Authors:** Federica Sacco, Bruno Paun, Oriol Lehmkuhl, Tinen L. Iles, Paul A. Iaizzo, Guillaume Houzeaux, Mariano Vázquez, Constantine Butakoff, Jazmin Aguado-Sierra

**Affiliations:** ^1^Barcelona Supercomputing Center (BSC), Barcelona, Spain; ^2^PhySense, ETIC, Universitat Pompeu Fabra, Barcelona, Spain; ^3^Visible Heart Laboratory, Department of Surgery, University of Minnesota, Minneapolis, MN, United States; ^4^IIIA - CSIC, Bellaterra, Spain

**Keywords:** trabeculae, papillary muscles, left ventricular modeling, left ventricular hemodynamics, porosity

## Abstract

The aim of the present study is to characterize the hemodynamics of left ventricular (LV) geometries to examine the impact of trabeculae and papillary muscles (PMs) on blood flow using high performance computing (HPC). Five pairs of detailed and smoothed LV endocardium models were reconstructed from high-resolution magnetic resonance images (MRI) of *ex-vivo* human hearts. The detailed model of one LV pair is characterized only by the PMs and few big trabeculae, to represent state of art level of endocardial detail. The other four detailed models obtained include instead endocardial structures measuring ≥1 mm^2^ in cross-sectional area. The geometrical characterizations were done using computational fluid dynamics (CFD) simulations with rigid walls and both constant and transient flow inputs on the detailed and smoothed models for comparison. These simulations do not represent a clinical or physiological scenario, but a characterization of the interaction of endocardial structures with blood flow. Steady flow simulations were employed to quantify the pressure drop between the inlet and the outlet of the LVs and the wall shear stress (WSS). Coherent structures were analyzed using the Q-criterion for both constant and transient flow inputs. Our results show that trabeculae and PMs increase the intra-ventricular pressure drop, reduce the WSS and disrupt the dominant single vortex, usually present in the smoothed-endocardium models, generating secondary small vortices. Given that obtaining high resolution anatomical detail is challenging *in-vivo*, we propose that the effect of trabeculations can be incorporated into smoothed ventricular geometries by adding a porous layer along the LV endocardial wall. Results show that a porous layer of a thickness of 1.2·10^−2^ m with a porosity of 20 kg/m^2^ on the smoothed-endocardium ventricle models approximates the pressure drops, vorticities and WSS observed in the detailed models.

## 1. Introduction

Computational cardiac modeling has become important as a non-invasive modality to study the overall cardiac function (Trayanova, 2011; Taylor et al., 2013). Recently, regulatory bodies are encouraging and supporting the use of *in-silico* modeling to reduce animal experimentation. Within this context, models of cardiac hemodynamics have yet to be improved. The majority of the hemodynamic cardiac computational simulations consider simplified geometries with smoothed endocardial surfaces (Doost et al., 2015; Khalafvand et al., 2015; Imanparast et al., 2016), mostly due to a lack of high-resolution, fast and safe *in-vivo* imaging techniques. It is also true that solving highly detailed models require computationally expensive simulations that can only be carried out using HPC.

In reality the heart anatomy is complex and all individuals have their own unique anatomies. The interior of the cardiac chambers is not smooth: it is populated by PMs, trabeculae of different sizes and false tendons (Gao et al., 2014). In the LV, PMs are the muscles responsible for properly positioning the chordae tendinae during systole to optimize mitral valve leaflet coaptation. Trabeculae are complex muscular structures that are unique to a given human heart, mostly consisting of myocytes, that protrude from the endocardial wall into the interior of the ventricle and present a sponge-like structure. The primary role of the trabeculae in the overall cardiac function remains unknown, but they are often associated with the Purkinje network.

State of the art LV CFD simulations, employing detailed endocardial structure models, have been created from either MRI or computed tomography (CT) *in-vivo* modalities (Chnafa et al., 2016; Lantz et al., 2016; Vedula et al., 2016), but they only incorporated PMs and a few large trabeculae. In previously reported studies, Chnafa et al. (2016) used 4D MR images to reconstruct the LV geometry, characterized only by PMs, and prescribe physiological deformations using numerical treatments. In this way the author could study blood flow instabilities within the ventricular cavity and found out that high-frequency flow fluctuations can be common in normal LVs. Both Vedula et al. (2016) and Lantz et al. (2016) added also few big trabeculae together with the PMs in their LV geometries and studied the impact of these endocardial structures on the blood flow by comparing simulations results with smoothed-endocardium ventricles. Vedula et al. (2016) reconstructed the LV geometry from high-resolution 4D CT scans and applied prescribed mesh deformation based on immersed boundary method. The authors observed a “scrambling” of blood flow vortices produced by PMs and trabeculae, which caused the generation of deeper and more complex vortices that were not present in the smoothed model. In this way, trabeculations help diastolic filling and, during systole, they help ventricular washout by wringing out the blood flow out from the apex. Lantz et al. (2016) extracted the LV endocardial surface and wall motion over time from 4D CT data. In contrast with Vedula et al. (2016) and Lantz et al. (2016) did not observe any deep penetration of the mitral inflow jet toward the apex: the jet strongly interacted with the PMs and was diverted toward the outflow tract. However, both papers have demonstrated that the detailed anatomies of LV endocardium have important influences on blood flow dynamics: in particular, particle tracking used by Lantz et al. (2016) demonstrated that blood flow interacted with trabeculae and PMs, creating vortices around the endocardial spaces between the trabeculations. More vortices appeared during diastole in the detailed LV as compared to the smoothed one, and PMs redirected blood flow and generated a large vortex, which was not present in the smoothed model. Finally, it was shown that the presence of trabeculations created a region where the flow appeared to be stagnant during five cardiac cycles, which is impossible to reproduce with smoothed endocardium models. While Vedula et al. (2016) and Lantz et al. (2016) considered the PMs and few trabeculae, the level of detail and the amount of trabecular structures in LV geometry reconstructions was not as high as in Kulp et al. (2011), who segmented detailed endocardial structures from high-resolution 4D CT data. The authors studied the interaction between trabeculations and the blood flow by deforming the initial 3D mesh in each following frame. Results showed how the complex endocardial surface caused the blood to move through the empty spaces between the trabeculations and fill these cavities during diastole.

In this paper we used highly detailed anatomical LV endocardium models to characterize the effects of trabeculae and PMs on the blood flows using CFD simulations. Four detailed LV geometries were reconstructed from high resolution imaging data of perfusion fixed human hearts (2 male and 2 female), which were obtained at the Visible Heart® Laboratory (Atlas of Human Cardiac Anatomy, RRID:SCR_015734). Detailed and smoothed endocardial models were reconstructed for each of the four hearts to quantify the differences between these two cases and thus characterize the impacts of PMs and trabeculae on ventricular hemodynamics. The level of detail in these reconstructions of the endocardial structures was, to the best of our knowledge, the highest ever achieved for this kind of study: the average size of the smallest structures reconstructed measures about 1 mm^2^ in cross-sectional area.

A fifth (male) LV geometry, named *control LV*, was reconstructed from the human hearts high resolution images dataset, together with its smoothed equivalent. This model was only characterized by PMs and few large trabeculae: in this way we could compare simulation results obtained from the highly detailed models described previously to the ones from an LV geometry which is similar in detail to those present in literature (Kulp et al., 2011; Chnafa et al., 2016; Lantz et al., 2016; Vedula et al., 2016).

Through CFD simulations we aim to characterize the hemodynamics inside detailed vs. smoothed human ventricular anatomies by quantifying the trabecular volume, intra-ventricular pressure drop, WSS and vorticity within the LV cavities. Furthermore, we propose that a porous layer can be added to the LV endocardium to compensate for the absence of trabeculae within smoothed ventricular hemodynamic models. Our main findings show that the presence of trabeculae alters significantly the blood flow by increasing intra-ventricular pressure drop, reducing the shear stress at the ventricular walls and generating multiple secondary vortices, absent in smooth-walled ventricle simulations. Furthermore, our results show that indeed a porous layer can compensate for the absence of trabeculations inside simplified ventricular models by increasing the intra-ventricular pressure drop, by reducing the wall shear stress at the interface of the porous layer and by increasing the amount of vortex structures within the LV.

## 2. Materials and methods

### 2.1. Left ventricular models

The five LV models used in this work were reconstructed from high-resolution MR images obtained from *in vitro* perfusion of fixed human hearts. The research uses of these heart specimens have received appropriate approval from both the University of Minnesota's Institution Review Board and LifeSource Research Committee (Minnesota's non-profit procurement donation organization). The hearts were recovered from organ donors whose hearts were not viable for transplantation. Written and informed consents were obtained from the donors families which follow the wishes of the donor. The database is open to public access.

**Figure 1:**
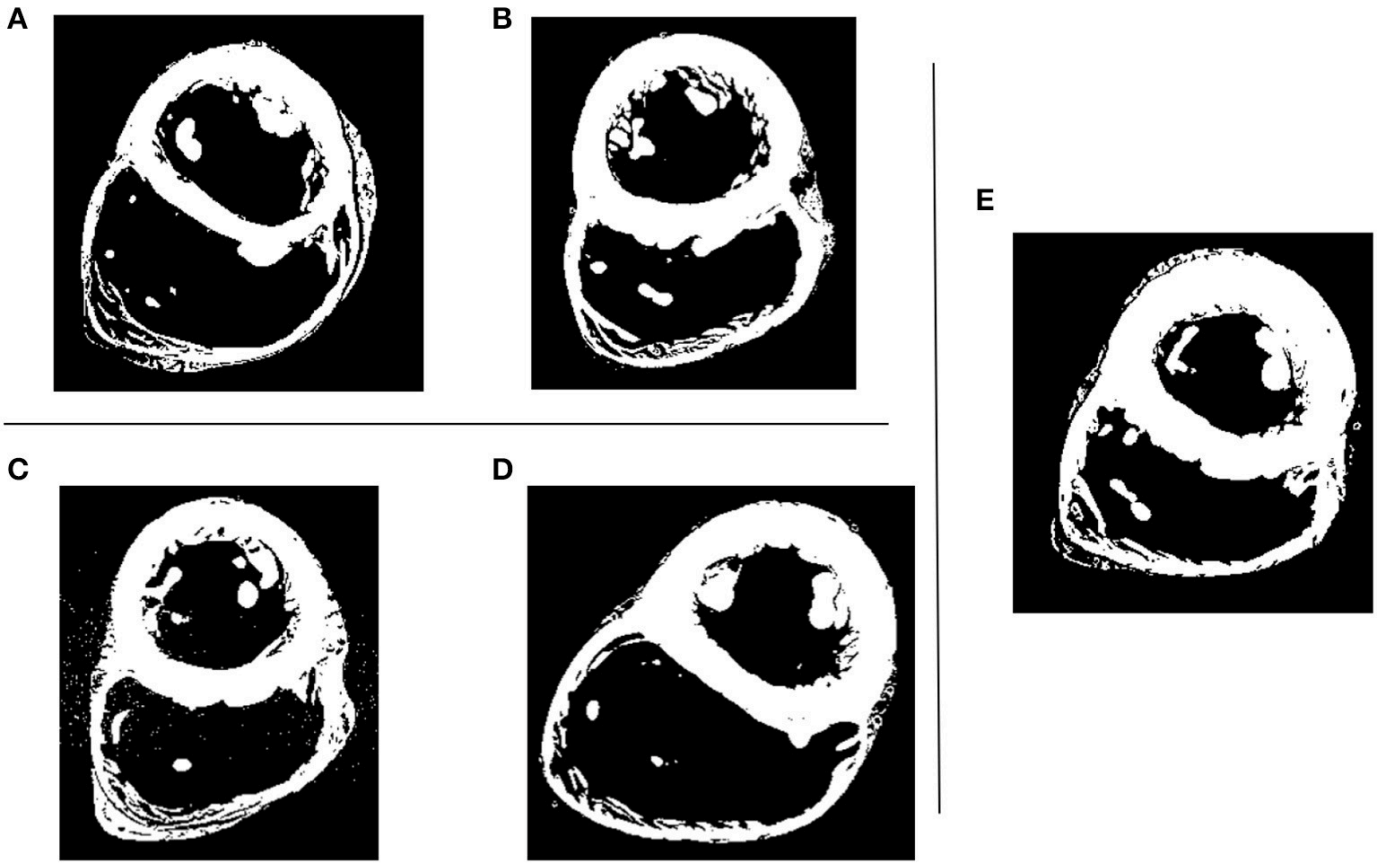
Five human hearts high resolution MRI datasets. Shown is the short axis view at mid-cavity height. **(A–D)** LV models with smoothed (left) and detailed (right) endocardial surfaces along with the *control LV* **(E)**.

DICOM data sets were acquired utilizing a 3T Siemens scanner with 0.44 × 0.44 mm in-plane resolution and slice thickness of 1 to 1.7 mm. The hearts were fixed with 10% formalin in phosphate buffered saline (PBS) solution for at least 24 h under 40–50 mmHg of pressure and then stored in 10% formalin. The five hearts DICOM datasets are shown in Figure 1.

Image segmentation was carried out with Fiji software (Fiji, RRID:SCR_002285), using the maximum entropy-based thresholding algorithm (Qi, 2014), followed by endocardial surface reconstruction using marching cubes algorithm in Seg3D (Seg3D, RRID:SCR_002552). The relative high contrast of the images guaranteed that the thresholding produced detailed endocardial models. The smoothed models were generated from the detailed geometries by manually deleting trabeculae and PMs and closing holes on associated surfaces using ReMESH software (ReMESH, RRID:SCR_015735). Autodesk Meshmixer (Autodesk Meshmixer, RRID:SCR_015736) sculpting software was then used to adjust the smoothed endocardial surface as to maintain the same outline for both the smoothed and detailed geometries.

The *control LV*, as a representative of a state of the art anatomical model, was reconstructed using a regularized region growing algorithm of ITK-SNAP (ITK-SNAP Medical Image Segmentation Tool, RRID:SCR_002010) to get only large scale anatomical detail from the images. The algorithm allowed controlling the smoothness of the extracted contour making it easier to obtain the smoothed surface with just the PMs and a few large trabeculae (approximately 5 mm^2^ in cross-sectional area). The obtained level of detail for the *control LV* was similar to the reported models used in recent publications on blood flow analysis in LV such as Vedula et al. (2016) and Chnafa et al. (2016).

In order to let the flow develop, a 50 mm long tube was attached at the inlet (corresponding to the mitral valve orifice) and a 70 mm tube at the outlet (corresponding to the aortic valve orifice). Each tube base matched exactly the corresponding valvular ring plane. Tubes were created for every given LV model using ParaView (ParaView, RRID:SCR_002516).

The resulting surface meshes were uniformly remeshed using Remesh and then volumetric tetrahedral meshes were generated using an isosurface-stuffing-based algorithm (Labelle and Shewchuk, 2007) with an in-house mesher developed at the Barcelona Supercomputing Center (BSC). The volumetric meshes had adaptive element size, with volumes varying from 10^−7^ mm^3^ to 1.9·10^−2^ mm^3^, with an average size of 5.7·10^−5^ mm^3^. Wireframe zoomed images of the tetrahedral meshes can be found in Figure S1.

**Figure 2:**
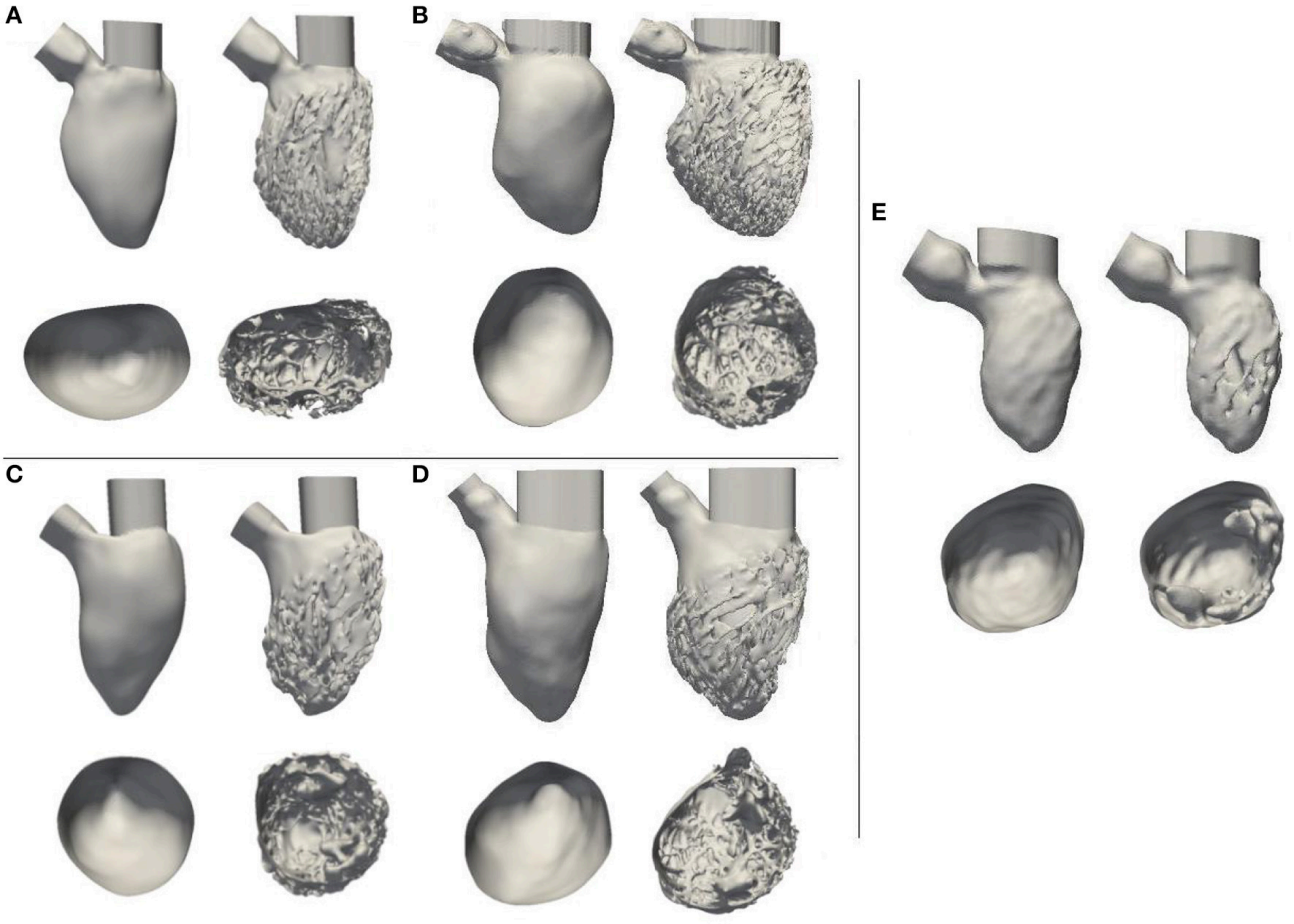
**(A–D)** LV models with smoothed (left) and detailed (right) endocardial surfaces along with the *control LV* **(E)**. Below each LV, the reciprocal ventricular cavity section, viewed from top, is shown.

The five LV anatomies, both smoothed and detailed, are shown in Figure 2. A more detailed view is reported in Figure S2. The medical histories and related information can be found in Table 1 of the Supplementary Materials. The ventricular volume of each mesh is reported in Table 2 of the Supplementary Materials.

### 2.2. Hemodynamic simulations

To carry out CFD simulations the walls were defined as rigid, no-slip boundary conditions. For the outlet, a stabilizing boundary condition employed a baseline pressure of 10.7 kPa (80 mmHg, a normal end-diastolic arterial pressure) plus an outflow resistance (Bazilevs et al., 2009). Blood viscosity was set to 0.0035 kg/(m · s) and density to 1,060 kg/m^3^.

Hemodynamic simulations solving continuity and Navier-Stokes equations for incompressible flows were run on the MareNostrum 4 supercomputer (MareNostrum, RRID:SCR_015737) and on Archer (ARCHER, RRID:SCR_015854), UK supercomputer, using Alya, the BSC's in-house, parallel multi-physics, HPC solver (Houzeaux et al., 2009; Vazquez et al., 2016).

Simulations were carried out using a low dissipation finite element (FE) strategy described below. The Navier-Stokes equations for a fluid domain Ω bounded by Γ = ∂Ω within the time interval (*t*_0_, *t*_*f*_) reside in calculating a velocity **u** and a kinematic pressure *p* so that Equations (1, 2) are satisfied; where ν is the kinematic viscosity, **f** is the vector of external body forces and **S**(**u**) is the rate-of-strain tensor.

(1) $$\partial_{t}u+(u\cdot \nabla )u-2\nu \nabla \cdot S(u)+\nabla p-f=0\text{ in}\;\Omega \times (t_{0},t_{f})$$

(2) $$\nabla \cdot u=0\text{ in}\;\Omega \times (t_{0},t_{f})$$

To obtain weak or variational formulation of the Navier-Stokes equations Equations (1, 2), we introduced the spaces of vector functions $V_{D}=H_{D}^{1}\left(\Omega \right)$, $V_{0}=H_{0}^{1}\left(\Omega \right)$ and $Q=L^{2}\left(\Omega \right)/ℜ.$. *L*^2^(Ω) is the space of square-integrable functions, *H*^1^(Ω) is a subspace of *L*^2^(Ω) formed by functions whose derivatives belong also to *L*^2^(Ω), $H_{D}^{1}\left(\Omega \right)$ is a subspace of *H*^1^(Ω) which satisfies Dirichlet boundary condition on Γ. $H_{0}^{1}\left(\Omega \right)$ is a subspace of *H*^1^(Ω) whose functions are zero on Γ; and ${\text{H}}_{D}^{1}\left(\Omega \right)$ and ${\text{H}}_{0}^{1}\left(\Omega \right)$ are their vector counterparts in a two- or three-dimensional space. (·, ·) determines the standard *L*^2^ inner product. For the evolutionary case, $V_{t}\equiv L^{2}\left(t_{0},t_{f};V_{D}\right)$ and $Q_{t}\equiv D^{\prime }\left(t_{0},t_{f};Q\right)$ were introduced, where $L^{p}\left(t_{0},t_{f};X\right)$ is the space of time dependent functions in a normed space *X* so that $\int_{t_{0}}^{t_{f}}{\left\|f\right\|}_{X}^{p}\text{ d}t<\infty$, 1 ≤ *p* < ∞ and *Q*_*t*_ consists of mappings whose *Q*-norm is a distribution in time. The weak form of problem (Equations 1, 2) with the boundary conditions is then: Find **u** ∈ **V**_*t*_, *p* ∈ *Q*_*t*_ such that Equation (3) is satisfied for every (**v**, *q*) ∈ **V**_0_ × *Q*.

(3) $$\begin{array}{l} \left(\partial_{t}u,v\right)+\left(u\cdot \nabla u,v\right)+2\nu (Su,\nabla v)-\left(p,\nabla \cdot v\right)+\left(q,\nabla \cdot u\right)\\ \;-\left(f,v\right)=0, \end{array}$$

Moreover, in the previous equations the non-linear term convective form reported in Equation (4) was used, which is the most frequent choice in computational practice. Using Equation (2), other non-linear term forms can be derived, which are the same at the continuous level but do have different properties at the discrete level. In Equation (5) we consider the energy, momentum and angular momentum conserving form recently proposed in Charnyi et al. (2017). A non-incremental fractional-step method was used for pressure stabilization. This allows the use of finite element pairs which do not satisfy the inf-sup conditions, like the equal order interpolation for the velocity and pressure used in this work. An energy conserving Runge-Kutta explicit method lately proposed by Capuano et al. (2017) along with an eigenvalue based time-step estimator (Trias and Lehmkuhl, 2011) were used in order to time integrate the set of equations. This methodology, recently proposed by Lehmkuhl et al. (2017), follows the principles of Verstappen and Veldman (2003), generalized for unstructured finite volumes by Jofre et al. (2013) and Trias et al. (2014) but in a FEM framework. The presented methodology has been successfully validated and benchmarked vs. other popular CFD approaches and experimental data in the bioengineering flows environment in Koullapis et al. (2017).

(4) $$NL_{conv}\left(u\right)=u\cdot \nabla u$$

(5) $$NL_{emac}\left(u\right)=2S\left(u\right)u+\left(\nabla \cdot u\right)u$$

**Figure 3:**
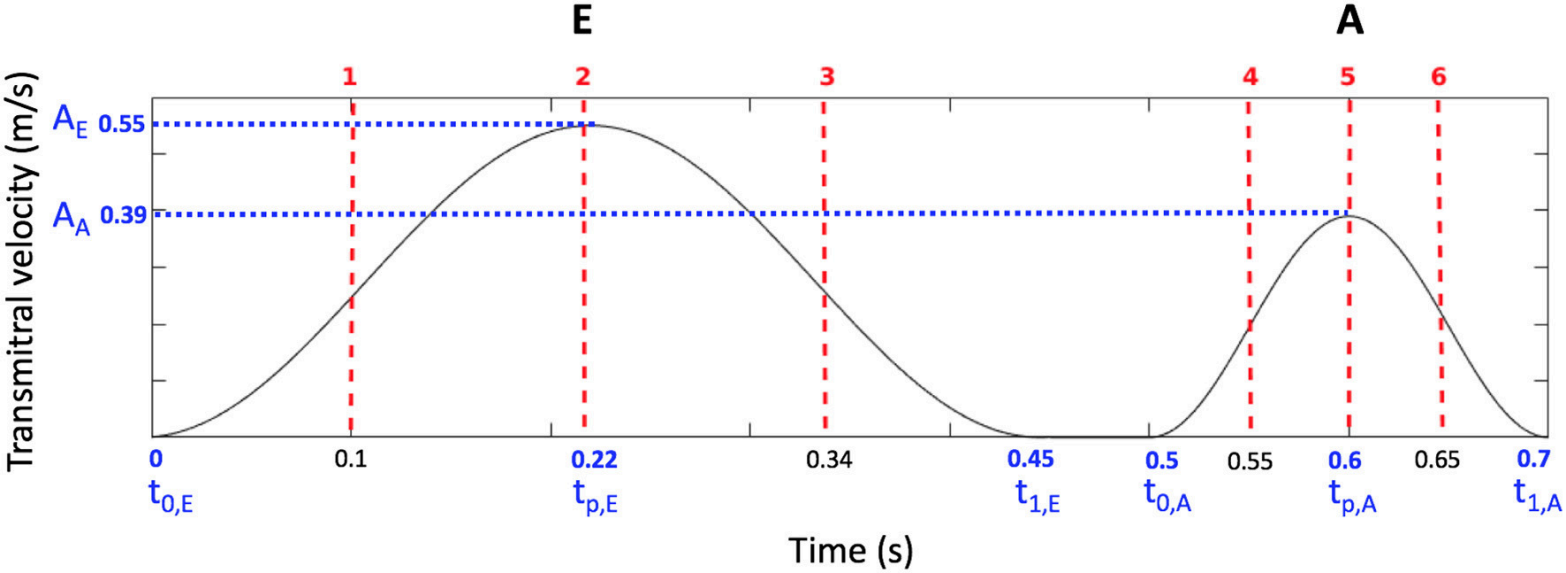
Synthetic transmitral E-A wave input function, wave parameters (blue) and the six time instants shown in Figure 10 (red).

**Figure 4:**
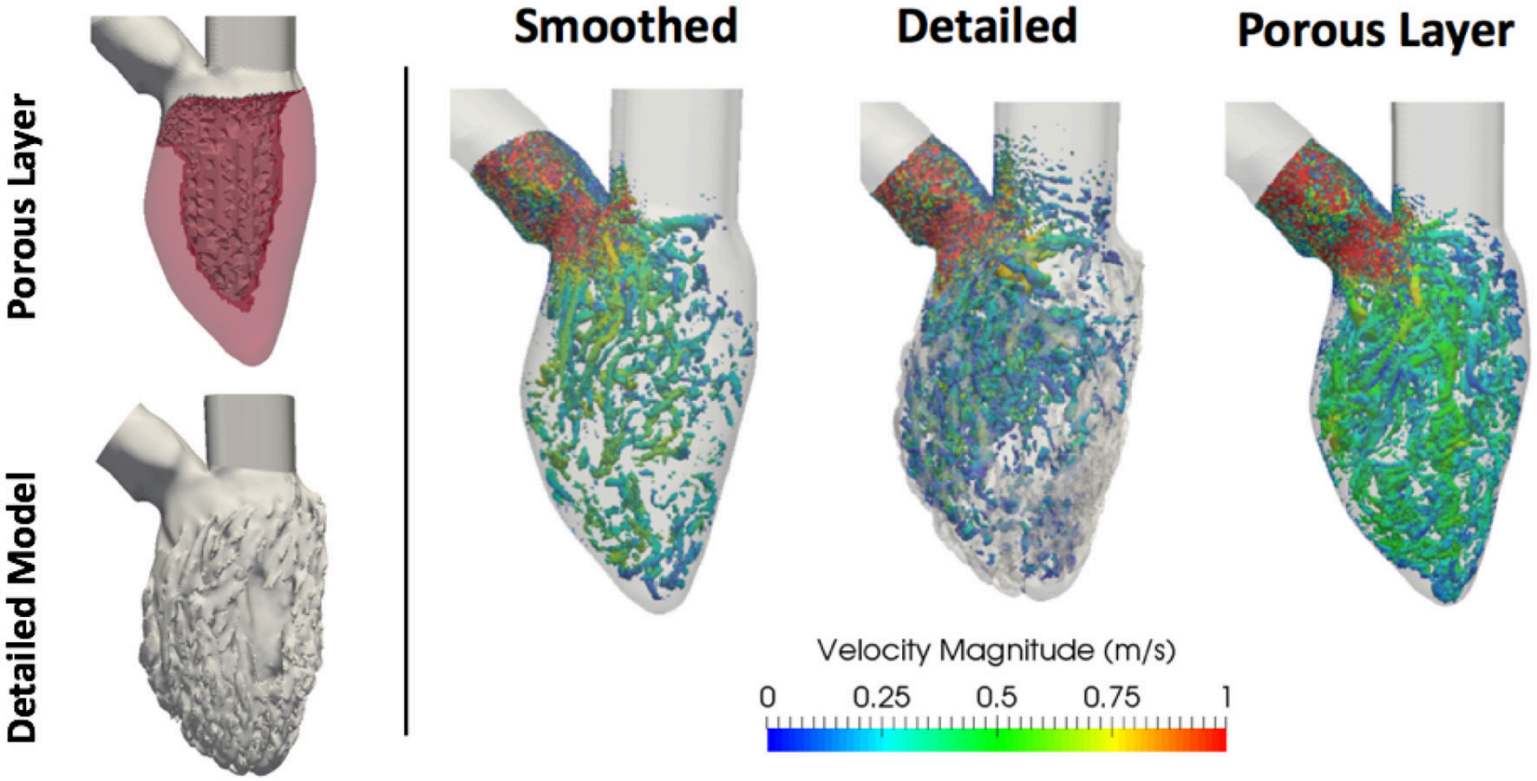
**(Left)** Porous layer (in light red) on subject A LV wall and corresponding detailed model. **(Right)** Vorticity estimated using the Q-criterion, thresholded at 5,000 s^−2^, for constant inflow simulations with smoothed, detailed and smoothed with porous layer geometries. Vortices are colored by velocity magnitude [m/s].

We performed the following simulations:

1. *Constant inflow*. This was done to characterize purely the influences of the geometries on the hemodynamics. A constant, flat-profile, blood flow velocity of 0.55 m/s (peak normal transmitral inflow velocity Fernández-Pérez et al., 2012) was applied at the inlet of all models.
2. *Synthetic E-A wave mitral valve inflow*. A synthetic E-A wave was created as a combination of two cosine functions as in Equation (6). *A* was the maximum amplitude of the wave, *t*_0,*E*_, *t*_0,*A*_, and *t*_1,*E*_, *t*_1,*A*_ were the initial and final time of E and A waves respectively. *t*_*p,E*_ and *t*_*p,A*_ were the time corresponding to the peaks of E and A waves and *t* was the simulation duration. The parameters of the wave were chosen to represent a standard E-A wave of a healthy individual (Fernández-Pérez et al., 2012); their values and the corresponding function are shown in Figure 3.
3. *Constant inflow with a porous layer*. A layer with the properties of a uniform porous material was added to the interior walls of the smoothed-endocardium models (see Figure 4). The motivation is that a porous layer is easy to implement and may provide similar hemodynamic characteristics than the irregular endocardial wall. The initial permeability value considered was the minimum of well graded gravel (5·10^−4^ m/s), that yields a porosity of 7 kg/m^2^ (Standard, 1999). The thickness and permeability of the porous material were empirically selected for model A so that the smoothed-endocardium model with this layer generates similar intra-ventricular pressure drop as the corresponding detailed model. In order to solve the CFD with the porous material, the momentum conservation equation was stated in the form of Darcy's law (Equation 6), where μ is the dynamic viscosity, *k* is the permeability, *u* is the velocity and *p* is the pressure, and added to the Navier-Stokes equations. To find the optimal combination of thickness and porosity of the porous layer, we did a sensitivity analysis varying the thickness from 1·10^−2^ to 1.2·10^−2^ m with porosities of 7, 20, 40, and 70 kg/m^2^. The parameters of the porous layer selected for model A was applied to models B, C, and D to verify if a single porous layer approximation can reproduce the hemodynamic behavior in the rest of the models.

(6) $$v\left(t\right)=\{\begin{array}{ll} \frac{A_{E}}{2}\left(1+\cos\left(\frac{2\pi \left(t-t_{p,E}\right)}{t_{1,E}-t_{0,E}}\right)\right) & t_{0,E}\leq t\leq t_{1,E}\\ 0 & t_{1,E}<t<t_{0,A}\\ \frac{A_{A}}{2}\left(1+\cos\left(\frac{2\pi \left(t-t_{p,A}\right)}{t_{1,A}-t_{0,A}}\right)\right) & t_{0,A}\leq t\leq t_{1,A} \end{array}$$

(7) $$u=-\frac{1}{\mu }k(\nabla p)$$

### 2.3. HPC characteristics

HPC characteristics for both constant and transient inflow simulations in terms of cores, total simulation time and time step are reported in Tables 1, 2. Every simulation, with both constant and transient inflow, was run up to 800 ms. Information on the scalability of the incompressible flow module within Alya multi-physics solver can be found in the works of Houzeaux et al. (2009) and Vazquez et al. (2016). The elements-per-core ratio that was used to run these hemodynamic simulations was about 25,000.

**Table 1:** Constant inflow simulations results and HPC information on both constant and transient inflow simulations.

**Hearts**	**A**		**B**		**C**		**D**		**E**	
**Geometry**	**Smoothed**	**Detailed**	**Smoothed**	**Detailed**	**Smoothed**	**Detailed**	**Smoothed**	**Detailed**	**Smoothed**	**Detailed**
Area inlet [m^2^]	6 · 10^−4^		10.2 · 10^−4^		5.7 · 10^−4^		9.4 · 10^−4^		13.9 · 10^−4^	
Area outlet [m^2^]	2.5 · 10^−4^		2.4 · 10^−4^		3 · 10^−4^		1.8 · 10^−4^		4.9 · 10^−4^	
α [degrees]	49		72.2		58.8		43.3		73.7	
*d* [m]	2.1 · 10^−2^		1.9 · 10^−2^		2.3 · 10^−2^		2.3 · 10^−2^		2.3 · 10^−2^	
Trabeculae [%]	21.4		26.6		19.6		21.3		10.9^a^	
Volumetric mesh elements	3,012,240	2,329,175	3,545,080	5,525,858	1,773,680	5,039,205	1,588,160	3,251,790	2,583,680	4,272,960
Volumetric mesh points	562,926	476,951	632,642	1,140,117	333,305	1,038,890	204,189	671,442	541,204	764,903
Reynolds number at inlet	6,004		4,605		4,477		5,764		7, 009	
ΔP [kPa]	1.3 ±0.1	1.5 ±0.1	3.8 ±0.04	4.1 ±0.1	0.7 ±0.06	0.8 ±0.05	2.2 ±0.1	4.9 ±0.2	2.3	2.5 ±0.1
Δ*P*_*diff*_ [kPa]	0.2		0.3		0.1		2.7		0.2	
WSS mode [Pa]	0.35	0.05	0.06	0.05	0.75	0.05	0.45	0.05	0.15	0.25
WSS median [Pa]	0.51	0.39	1.02	0.34	1.23	0.67	0.63	0.66	0.84	0.52
Total vortex surface [m^2^]	29.1 · 10^−3^	35.6 · 10^−3^	33.8 · 10^−3^	60.4 · 10^−3^	20.4 · 10^−3^	37.7 · 10^−3^	45 · 10^−3^	48.4 · 10^−3^	29.4 · 10^−3^	35.3 · 10^−3^
Cores	144	96	144	240	96	240	96	144	144	192
C.I.^b^ Simulation time [hh:mm]	63:47	254:50	91:13	110:10	41:43	134:18	18:17	419:47	154:15	113:37
C.I. Initial time step [s]	9.85 · 10^−05^	7.74 · 10^−05^	5.78 · 10^−05^	6.68 · 10^−06^	7.17 · 10^−05^	6.90 · 10^−05^	1.90 · 10^−04^	5.51 · 10^−05^	1.35 · 10^−04^	9.42 · 10^−05^
T.I.^c^ Simulation time [hh:mm]	81:25	167:52	335:12	340:18	336:33	53:50	265:20	213:53	17:20	120:46

*P, mode of the pressure distribution at inlet and outlet*.

*ΔP, intra-ventricular pressure drop from inlet to outlet*.

*ΔP_diff_ = ΔP_d_ − ΔP_s_, differences between detailed and smoothed pressure drops*.

a *In the case of the control LV we calculated the PMs volume, since this LV geometry was only characterized by PMs*.

b *C.I., Constant Inflow simulations*.

c *T.I., Transient Inflow simulations*.

**Table 2:** Constant inflow simulations results for A-D LVs with the porous layer of thickness 1.2 · 10^−2^ m and porosity 20 kg/m^2^, along with the corresponding HPC information.

**Hearts**	**A**	**B**	**C**	**D**
ΔP [kPa]	1.5 ± 0.04	3.9 ± 0.4	0.78 ± 0.04	2.6 ± 0.3
WSS mode [Pa]	0.003	0.47	0.23	0.25
WSS median [Pa]	0.36	0.32	0.51	0.55
Total vortex surface [m^2^]	36.8 · 10^−3^	61.3 · 10^−3^	40 · 10^−3^	54.6 · 10^−3^
Cores	144	144	96	96
Simulation time [hh:mm]	46:51	47:30	47:30	47:30
Initial time step [s]	5.5 · 10^−05^	6.39 · 10^−06^	7.68 · 10^−06^	2 · 10^−06^

*P, mode of the pressure distribution at inlet and outlet*.

*ΔP, intra-ventricular pressure drop from inlet to outlet*.

*ΔP_diff_ = ΔP_d_ − ΔP_s_, differences between detailed and smoothed pressure drops*.

### 2.4. Geometric markers

The following geometrical markers were used:

*Trabecular volume* was calculated as a difference between the volume of the convex hull of the detailed-endocardium LV model and the volume of the model itself.

**Figure 5:**
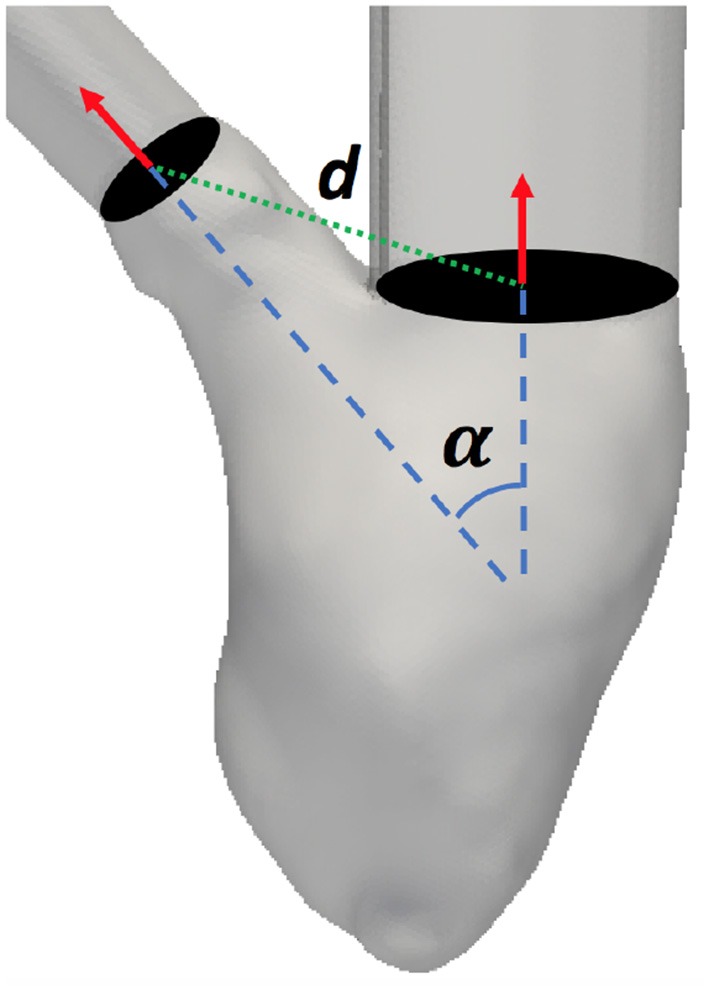
Angle (α) and distance (*d*) between the mitral and aortic valvular planes definition.

*The angle between inlet and outlet* was the angle between the vectors normal to the two valvular planes (mitral and aortic). An illustration can be seen in Figure 5.

*The distance between inlet and outlet* was the distance between the mitral and aortic valves centers. An illustration can be seen in Figure 5.

### 2.5. Hemodynamic analysis

#### 2.5.1. Intra-ventricular pressure drop

**Figure 6:**
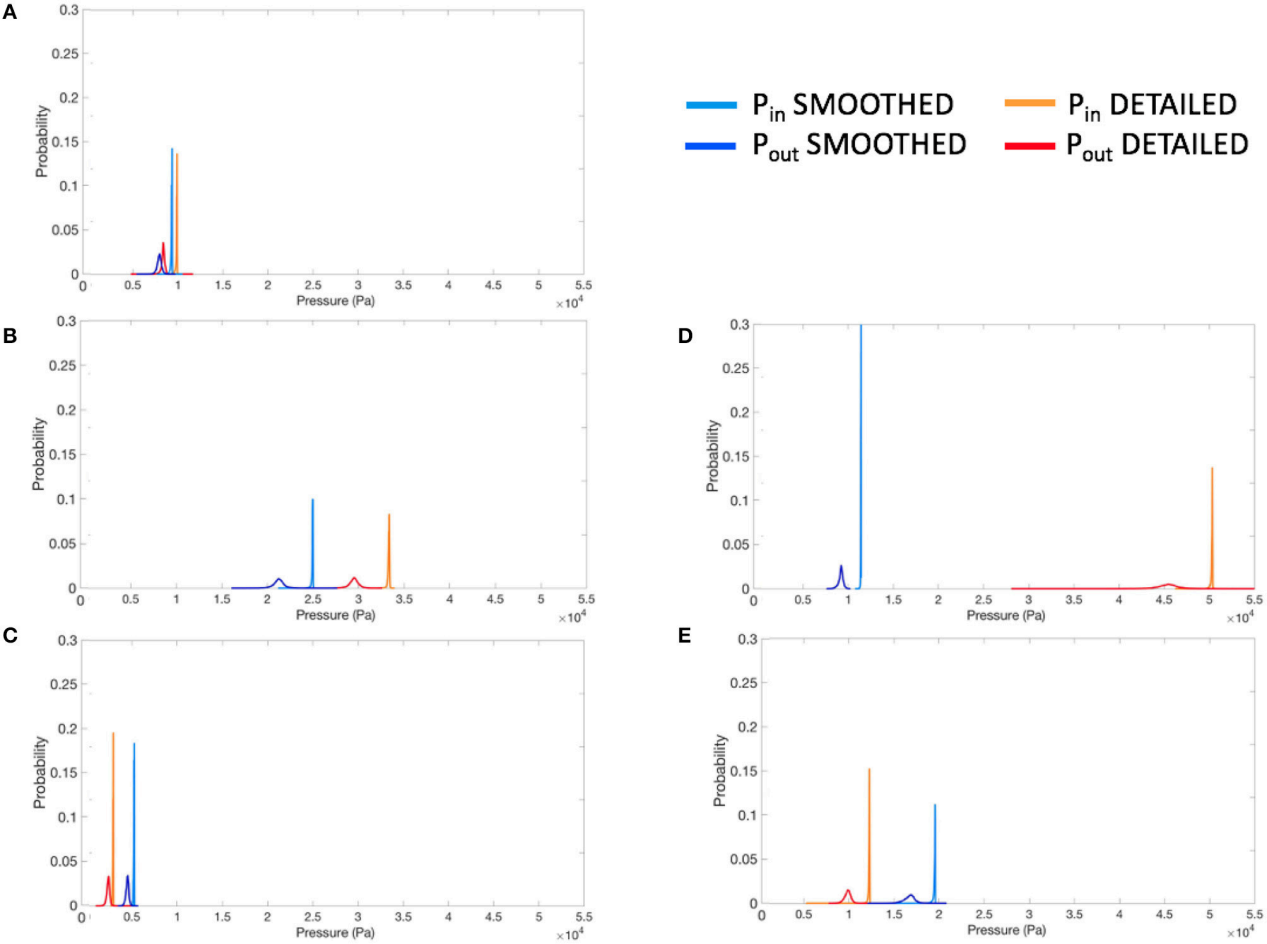
Normalized pressure histograms for each smoothed and detailed LV pair, at inlet (*P_in_*) and outlet (*P_out_*), for constant inflow simulations. **(A–D)** LV models with smoothed (left) and detailed (right) endocardial surfaces along with the *control LV* **(E)**.

The pressure distributions were analyzed within 15 mm long volumes of both inlet and outlet tubes. The sections were chosen right at the inlet of the mitral and at the outlet of the aortic valves. The histograms were normalized to unit area under the curve and bin width was calculated using the Freedman-Diaconis rule. As the pressure distributions were non-Gaussian (Figure 6), the intra-ventricular pressure drop was calculated as the difference between the inlet and outlet pressure mode. The pressure difference was then averaged over the ten last time frames (approximately 50 ms of simulations), during steady flow. From these results, we calculated the intra-ventricular pressure drop difference (Δ*P*_*diff*_) as the difference of the detailed and smoothed pressure drops for every studied LV.

#### 2.5.2. WSS on the ventricular walls

**Figure 7:**
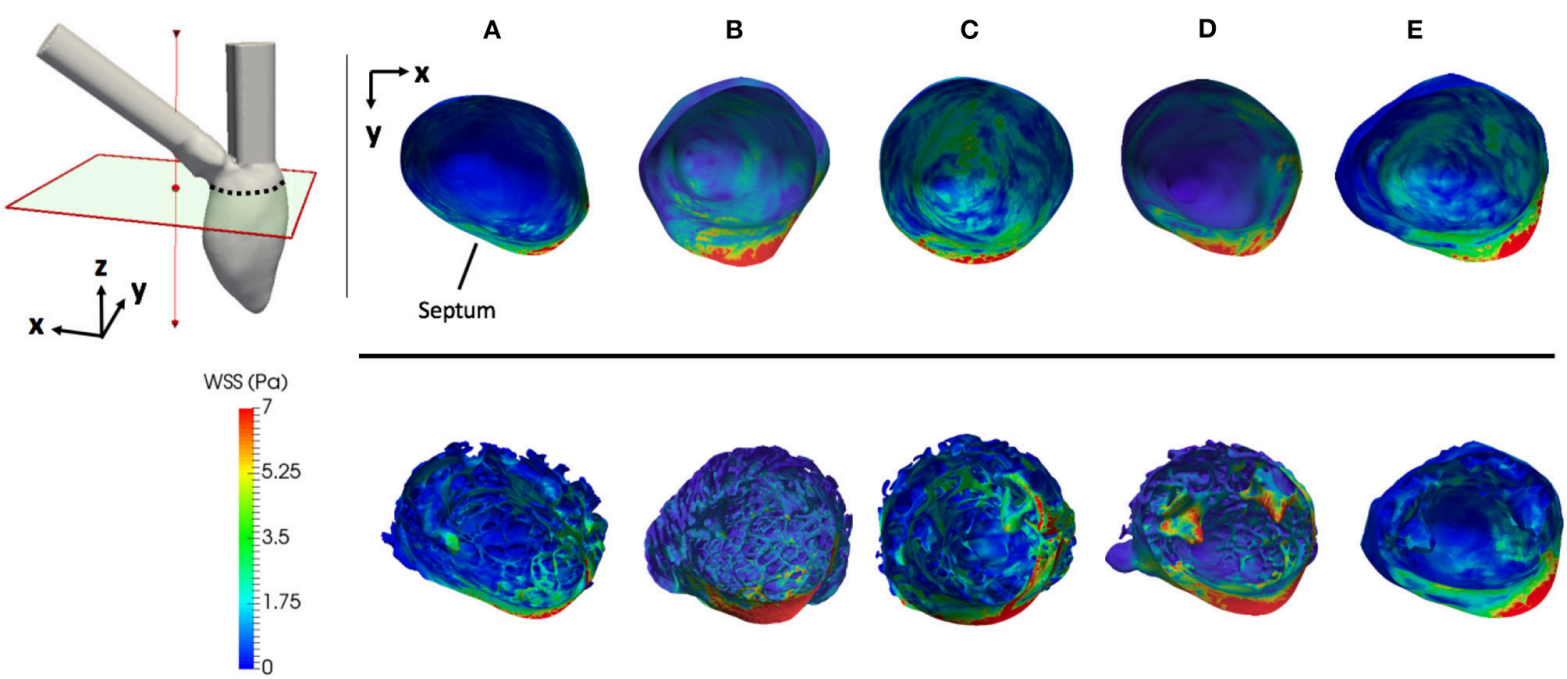
Magnitude of the WSS during constant inflow simulations on all cases. All geometries are clipped and aligned to the upper left axes in the figure. The septum is pointed out for spatial reference. **(A–D)** LV models with smoothed (left) and detailed (right) endocardial surfaces along with the *control LV* **(E)**.

WSS histograms were computed for every LV cavity (Figure 7) and normalized using the Freedman-Diaconis rule to choose the width of the bins. Given that these distributions were upward skewed, the median was used to analyze the WSS of each model. The total magnitude range and the mode are also reported for each case. In the case of the porous layer simulations, the median and mode WSS were calculated on the interface between the porous layer and the blood flow.

#### 2.5.3. Vorticity

Coherent structures were analyzed in both steady and transient inflow simulations applying the Q-criterion method (Hunt et al., 1988; Chakraborty et al., 2005). The applied thresholds for vortex visualization were 5,000 s^−2^ and 1,000 s^−2^ for steady and transient inflow simulations respectively. Vortex quantification was done in Paraview, by integrating the contours of the vortices to estimate the total surface area.

## 3. Results

### 3.1. Constant inflow

The results of constant inflow quantification for the five LVs are shown in Table 1. In all LVs, the intra-ventricular pressure drops (Δ*P*_*diff*_) increased in the detailed geometries by an average 0.2 kPa, except for subject D, that elicited the highest Δ*P*_*diff*_ of 2.7 kPa. Also the detailed E LV, exhibited a similar pressure drop to models A, B, and C, regardless of the low percentage of trabecular volume within its geometry. The geometrical markers do not indicate a direct correlation to the pressure drop (see Table 1). The pressure drop in models A, B, C, and E correlated best with the distance between their inlet and outlet, however, when model D is included, any good correlation disappears. The pressure drop is not correlated either to the Reynolds number at the inlet of each model.

**Figure 8:**
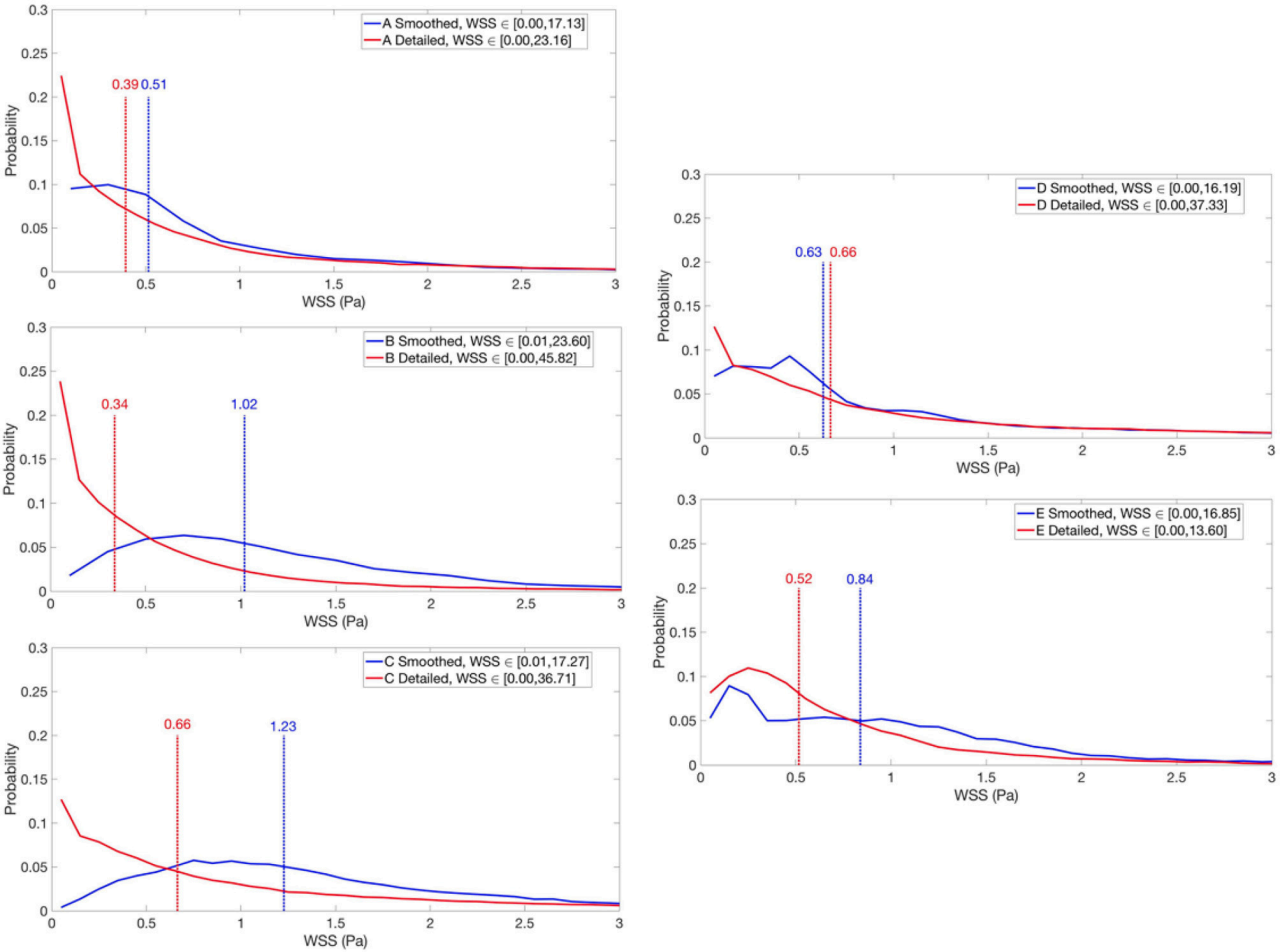
Normalized WSS histograms for smoothed and detailed LVs cavities under constant inflow. WSS medians are indicated with dashed lines. The range 0–3 Pa is highlighted for visualization purposes, but the maximum WSS values are included in each plot. (A–D) LV models with smoothed (left) and detailed (right) endocardial surfaces along with the *control LV* (E).

The magnitudes of the WSS for each case is shown in Figure 7. The WSS histograms are shown in Figure 8; they were cropped at 3 Pa for visualization purposes, but the maximum values are included in each plot. The median WSS decreased in the detailed geometries on all subjects, except for subject D, as shown in Table 1. Notice that in model D, in Figure 7, high WSS regions are markedly localized on the PMs and the outflow region. In model D the WSS median values remain relatively similar between the smoothed and detailed models, even though the mode values are significantly lower for the detailed geometry. It is important to point out that the peak WSS was higher on the detailed models in comparison with the smoothed geometries, except for model E.

**Figure 9:**
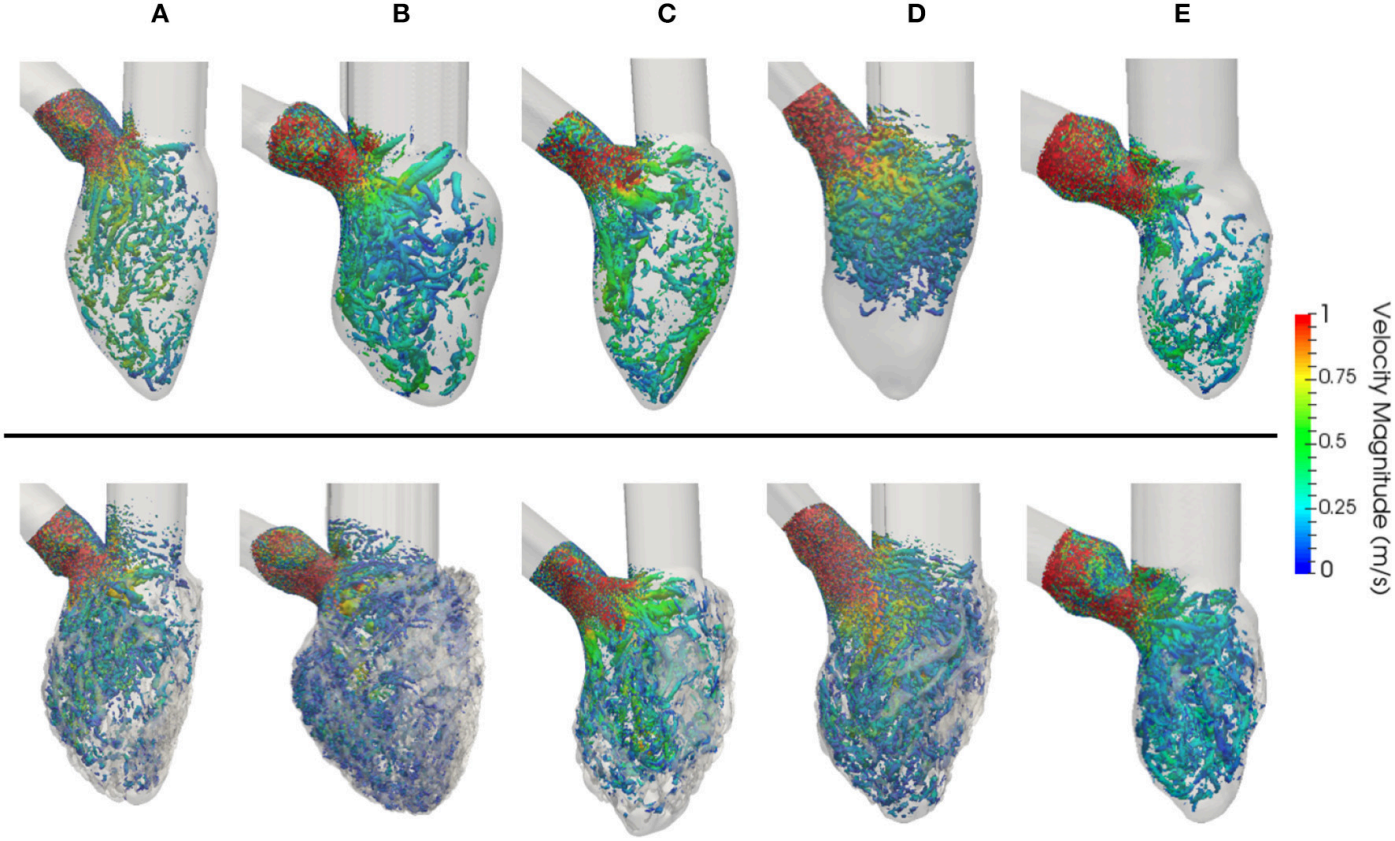
Vorticity estimated using the Q-criterion, thresholded at 5,000 s^−2^, for constant flow simulations in LVs with smoothed **(Top)** and detailed **(Bottom)** geometries. Vortices are colored by velocity magnitude [m/s]. **(A–D)** LV models with smoothed (left) and detailed (right) endocardial surfaces along with the *control LV* **(E)**.

The vortical structures shown in Figure 9 are thresholded at 5,000 s^−2^ and color coded according to the velocity magnitude [m/s]. The smoothed geometries generated fewer and larger vortices, while the detailed LVs showed the disruption of larger structures breaking down into a multitude of small scale vortices. To quantify them, the vortex contour surface area was calculated (shown in Table 3). The total surface area of the vortices was smaller in the smoothed (with a mean of 31.6 ± 8.9·10^−3^ m^2^) compared to the detailed LVs (with a mean of 43.5 ± 10.8·10^−3^ m^2^).

**Table 3:** Total vortex surface [m^2^] for the six instants (1−6) of the synthetic E-A wave in subject D.

**Geometry**	**1**	**2**	**3**	**4**	**5**	**6**
Smoothed	9.4 · 10^−3^	16.7 · 10^−3^	17.4 · 10^−3^	25.5 · 10^−3^	22.5 · 10^−3^	23.7 · 10^−3^
Detailed	14.9 · 10^−3^	18.1 · 10^−3^	24.4 · 10^−3^	29.2 · 10^−3^	25.9 · 10^−3^	26.6 · 10^−3^

### 3.2. E-A wave mitral valve inflow results

**Figure 10:**
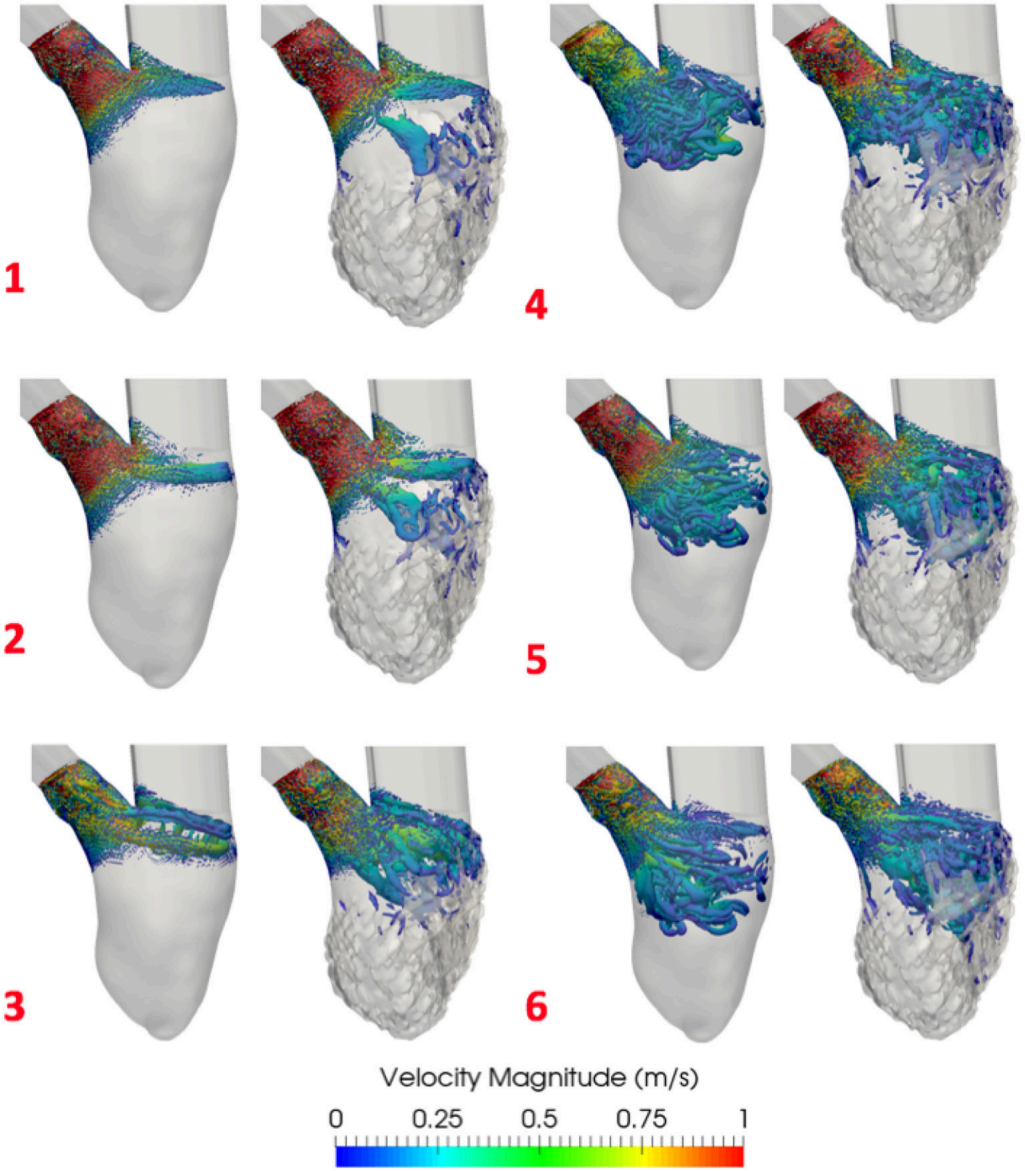
Vortices comparison in E-A wave inflow simulations with smoothed **(Left)** and detailed **(Right)** LV (D). Shown is the Q-criterion thresholded at 1,000 s^−2^. Vortices are colored by velocity magnitude [m/s]. 1–6 are the time instants selected from the EA wave represented in Figure 3.

From the E-A wave transmitral inflow function 6 time instants were selected, as highlighted in Figure 4: early E wave (1), E wave peak (2), late E wave (3), early A wave (4), A wave peak (5) and late A wave (6). Figure 10 shows the vortices in model D. The Q-criterion values were thresholded at 1,000 s^−2^ and colored according to the velocity magnitude [m/s]. Figure 10 and Table 3 show that the presence of trabeculae created secondary vortices at the early E wave (time instants 1), increasing the total vortex surface from 9.4·10^−3^ m^2^ in the smoothed to 14.8·10^−3^ m^2^ in the detailed geometry. The secondary vortices in the detailed LV penetrated deeper between the trabeculations during the late E wave (time instant 3). During the early and peak A wave, a second weaker vortex ring was formed and it mixed with the vortices generated during the early filling (time instants 4–5). Here, for both smoothed and detailed geometries, the total surface of the vortices increased due to the mixing vortices but the amount was still higher in the detailed compared to the smoothed LV (25.5 − 22.5·10^−3^ m^2^ vs. 29.2 − 25.9·10^−3^ m^2^ respectively).

### 3.3. Constant inflow with a porous layer

The proposed porous layer produces energy dissipation, which increases the intra-ventricular pressure drop and adds complexity to the blood flow. The results of the sensitivity analysis of the effect on the intra-ventricular pressure drops for each thickness-porosity combination are reported in Table 4. By adding a layer of 1.2·10^−2^ m thickness and a porosity of 20 kg/m^2^ to the smoothed subject A, we obtained the intra-ventricular pressure drop equal to the one of its detailed case (1.5 kPa). Additionally, the vortex visualization using the Q-criterion thresholded at 5,000 s^−2^ demonstrates that the amount of vortices in the smoothed LV with the porous layer are similar to the ones in the detailed case (see Figure 3), presenting a total vortex surface area of 36.8·10^−3^ m^2^. This may indicate that the roughness interacts with the boundary layer as a sand-grain roughness does, without prioritizing any particular flow direction, being then the proposed porous layer model very effective. The presence of the porous layer could also approximate the WSS calculated for the detailed geometry A as can be observed in Table 2, providing a relative error of only 0.076.

**Table 4:** Porous layer sensitivity analysis results for subject A.

**Geometry**	**Porous layer**							
Porous layer thickness [m]	1 · 10^−2^				1.2 · 10^−2^			
Porosity [kg/m^2^]	7	20	40	70	7	20	40	70
ΔP [kPa]	1.46	1.45	1.45	1.44	1.56	1.5	1.47	1.45
WSS median [Pa]	1.3	1.26	1.11	1.1	1.27	1.32	1.2	1.18
Total vortex surface [m^2^]	38.5 · 10^−3^	36.4 · 10^−3^	32 · 10^−3^	31.4 · 10^−3^	36.3 · 10^−3^	36.8 · 10^−3^	35.7 · 10^−3^	32.9 · 10^−3^

*Constant inflow simulations*.

The same layer with a thickness of 1.2·10^−2^ m and a porosity of 20 kg/m^2^ was then applied to all the other cases (subjects B, C, D). In subject B and C, the presence of the porous layer increased the intra-ventricular pressure drop to values similar to the ones obtained within the detailed cases (see Table 2) providing a relative error of just 0.2 and 0.02 respectively. In subject D, the intra-ventricular pressure drop increased slightly with the presence of the porous layer, but in this case the thickness and porosity values of the porous layer were not able to reproduce the high values of pressure drop obtained inside the detailed model (relative error is 0.46). However, in all the models with the porous layer the WSS was reduced as shown in Table 2 with the biggest relative error being 0.23 in model D. This table also shows how in all the cases the total vortex surface increased with the presence of the porous layer by providing values slightly higher than those of the detailed geometries in all cases, with the maximum relative error being −0.12 for model D.

## 4. Discussion

LV endocardiums of humans present a highly trabeculated appearance, which is often ignored in ventricular hemodynamic studies. Even though a few studies have been done to analyze the effect of papillaries and trabeculae on blood flow, no study to our knowledge has ever included small trabeculae of cross-sectional area of 1 mm^2^.

In this study we focused on characterizing solely the effects of the geometries on the hemodynamics using CFD simulations. The fact that the walls are rigid makes it impossible to extapolate any of the findings to a clinical or physiologically relevant scenario. This study provides an engineering-like approach to a very complex biological system, by quantifying and characterizing the interaction between endocardial structures and blood flow and providing a potential model to include the effect of the complex structures within the heart without the need of segmenting an extremely complex structure and running large simulations every time. Therefore the absence of the mitral valve may constitute a limitation if we were drawing physiological conclusions, however it is not the case for the kind of study presented in this manuscript.

Given the different metrics analyzed, there appears to be no direct correlation between the volume of trabeculae and the intra-ventricular pressure drop. For the geometrical markers, subject D, for example, presented the highest Δ*P*_*diff*_ and is characterized by the smallest angle between the valvular planes (43.3°). We hypothesize that the Δ*P*_*diff*_ obtained in model D is a result of the location of the PMs, which were positioned right below the inlet, disturbing the blood flow at the inlet, which led to a higher energy dissipation, not observable in the other cases. There is no direct correlation between the angle or distance of inlet and outlet and the Δ*P*_*diff*_. It is clear that the existence of rugosities along the endocardial walls alter the hemodynamics by creating flow recirculation regions, vortex disruption into secondary vortices, which increase the energy dissipation, hence increasing the intra-ventricular pressure drop in complex ways. A key observation is that the location and orientation of the mitral and aortic valvular rings influence the direction of the flow, and hence, the high WSS visible either on trabeculated regions or on the PMs. We hypothesize that this observation is responsible for the high maximum WSS magnitude on the detailed geometries. Given the surface area of trabeculae or papillaries, WSS tends to be concentrated in small regions (Figure 7), increasing its maximum magnitude. This fact may have high implications on local tissue remodeling. However, regardless of the range of WSS, in Figure 8 it can be observed that in the smoothed geometries the mode WSS was of 0.5–1 Pa, while in detailed meshes the mode drops to approximately 0 Pa. PMs and trabeculae reduced the WSS in LV about 23.5–66.7%. Wall shear stress is an important parameter in biology in general. Mechano-transduction is an important mechanism in biology. Even though it is practically impossible to measure it in-vivo in the LV, and it has never been reported before, the fact that WSS is reduced in the presence of trabeculae may provide an insight of the reason of why such endocardial structures exist. The presence of trabeculae and PMs generated a multitude of secondary vortices that were not present in the smoothed geometries, as shown in Figure 9. The overall vortex area decreased in the smoothed LVs (Table 1), with a mean of 31.6 ± 8.9·10^−3^ m^2^. The presence of detailed endocardial structures increased the amount of vortices with a mean area of 43.5 ± 10.8·10^−3^ m^2^. Subject D was intriguing. The effect of trabeculae and PMs led to a higher WSS median. This is because this LV is characterized by large PMs (noticeable in Figures 1, 8), which led to higher WSS concentrated on the PMs. A 4.8% higher WSS was indeed observed in the detailed D case. We hypothesize that the high pressure drop in case D was due to the prominent PMs, which disturbed flow markedly, increasing the energy dissipation within the intra-ventricular volume, and thus, generating a high intra-ventricular pressure drop (see Figure S3). The analysis of more geometries is required to further understand and characterize the effect of endocardial structures on hemodynamics.

The results from the control subject E demonstrated that the presence of only the PMs led to a low Δ*P*_*diff*_ (0.2 kPa), however the main impact appears to be the WSS distributions in comparison with the more detailed geometries. In other words, having only PMs and a few big trabeculae does not significantly modify the WSS.

Using a transient inflows (E-A wave) allowed the study of vortex formations following physiological inputs. In the smoothed LV vortices were nominal and the generation of the vortex rings was clearly visible during the E waves (see the example in Figure 10). On the other hand, in the trabeculated ventricles, the vortex rings were disrupted, generating a multitude of secondary vortices. The vortex surface areas are provided in Table 3. It can be observed that the total surface areas of the vortices were larger in the trabeculated, in comparison to the smoothed geometry. Furthermore, in the anatomically detailed LV the secondary vortices penetrated deeper between the trabeculations during the late E wave (time instant 3). Notice that the A wave seems to produce higher vorticity in this model. We hypothesize that the reason for this is that we are starting our simulation with an organized zero flow all throughout the model. Recirculation within the cavity at zero flow (diastasis) would create higher vorticity in the second inflow wave. In the smoothed case vortices were more compact, while in the anatomically detailed LV there were multiple vortices that tended to be pushed toward the apex during the late A wave (time instant 6). This finding is in accordance with the results from the work of Vedula et al. (2016), in which the authors suggests that the observed behavior may help increasing LV washout. On the other side, the main vortex ring disruption and secondary vortices formation due to LV trabeculations was not seen in the work of Lantz et al. (2016), where they noticed that the presence of PMs and trabeculae generates a large vortex in the middle of the LV cavity.

Preliminary results from replacing the trabeculae with a porous layer show that it is possible to obtain an intra-ventricular pressure drop similar to those generated by the detailed endocardial models. Only for subject D, the intra-ventricular pressure drop was not as high as the one observed in the detailed model. This is due to the presence of big PMs right below the mitral valve inlet, which, as explained previously, highly disturbed the flow and increased the energy dissipation.

Moreover, the addition of a porous layer on the smoothed geometries helped to reduce the WSS median in all the models providing small relative errors 0.07, 0.05, 0.23, and 0.16 respectively. Again, for model D, the median WSS had a bigger relative error mostly due to the large impinging of flow on the PMs, which increase the WSS median for that specific case. Finally, the presence of the porous layer disrupted the main vortices into smaller ones, increasing the total vortex surface to values slightly higher than the observed in the detailed cases, however, the relative errors range from −0.1 to −0.12, therefore reproducing the hemodynamic behavior in terms of vorticity.

### 4.1. Limitations

The main limitation of this study is the use of CFD, without fluid-structure interaction (FSI), valves or moving walls. The lack of motion prevents us from comparing any measured value to in-vivo heart function, however, as was mentioned before, this study attempts to characterize solely the geometry effects on hemodynamics. Future work involves the use of FSI to compare our findings to in-vivo measurements. Another limitation is the small sample size, which limits the generalization and statistical significance of our results.

The generation of smoothed ventricles from the trabeculated ones provides a degree of variability in the geometries created. We removed the trabeculae keeping the overall shape of the ventricle and the volume unchanged. This however is observer dependent and requires a fair amount of user interaction. In models A–D, the smoothing procedure led to slightly smaller estimated LV volumes due to the elimination of the trabeculae. In the case of model E, the ventricular surface was primarily characterized by PMs, presenting just a few trabeculae, which led to a larger volume in the smoothed heart.

An important limitation is that valves were not considered in our simulations. The presence of the mitral valve will direct the blood flow jet to create impinging and this will have some influence on the interaction between the flow and the detailed endocardial structures. The valves have also been reported to create a vortex ring right below its leaflets, as observed in previous studies (Töger et al., 2012; Vedula et al., 2016), which is impossible to capture in this study.

## 5. Conclusions

The highly anatomically detailed LV models developed in this study present a level of geometric information that was never achieved before. The simulations performed highlight the differences between blood flow CFD simulations in detailed vs. smoothed human ventricular models. The presence of detailed structures increase the intra-ventricular pressure drop, create multiple secondary vortices and decrease the WSS within the LV cavity. The amount of trabeculations have no direct correlation with the Δ*P*_*diff*_, which was noted highest in the female LV D case. To the best of our knowledge, our study analyzed for the first time intra-ventricular pressure drops to investigate the effects of trabeculae and PMs on LV hemodynamic modeling. LV hemodynamics in detailed geometries are more complex than we anticipated, hence, a detailed study with more subjects is necessary and ongoing. Furthermore, our results confirm that neglecting detailed endocardial structures prevent computational models from recreating the complex blood flow behavior within the ventricles. Given that HPC simulations and high resolution MRI data are not always accessible, we propose that a simulated porous layer on the endocardial wall of smoothed LV models can potentially substitute the highly detailed geometries. Finally, we demonstrated that by adding a layer of 1.2·10^−2^ m thickness and 20 kg/m^2^ porosity to the smoothed cases we obtained pressure drops and WSS similar to the ones in the detailed LVs. The porous layer also increased the amount of secondary vortices, close to the amount observed inside the trabeculated models.

## 6. Resource identification initiative

Atlas of Human Cardiac Anatomy, RRID:SCR_015734.
MareNostrum Supercomputer, BSC, Barcelona, RRID:SCR_015737.
ARCHER, UK National Supercomputing Service, RRID:SRC_015854.
Fiji, RRID:SCR_002285.
Seg3D, RRID:SCR_002552.
ReMESH, RRID:SCR_015735.
ITK-SNAP Medical Image Segmentation Tool, RRID:SCR_002010.
Autodesk Meshmixer, RRID:SCR_015736.
Paraview, RRID:SCR_002516.

## Author contributions

FS: Conception, drafting, data analysis and interpretation of data. BP: Data preprocessing, data analysis. TI, PI: Acquisition of data and critical revision. CB, JA-S: Conception, drafting, analysis, critical revision. MV, GH, OL: Implementation of the solvers, critical revision.

### Conflict of interest statement

The authors declare that the research was conducted in the absence of any commercial or financial relationships that could be construed as a potential conflict of interest.

## Abbreviations

LV: Left Ventricle
PMs: Papillary Muscles
HPC: High Performance Computing
MRI: Magnetic Resonance Imaging
CFD: Computational Fluid Dynamics
WSS: Wall Shear Stress
CT: Computed Tomography
BSC: Barcelona Supercomputing Center
PBS: Phosphate Buffered Saline
FE: Finite Element
FSI: Fluid Structure Interaction.

## References

[B1] Bazilevs Y. Gohean J. R. Hughes T. J. R. Moser R. D. Zhang Y. (2009). Patient-specific isogeometric fluid-structure interaction analysis of thoracic aortic blood flow due to implantation of the Jarvik 2000 left ventricular assist device. Comput. Methods Appl. Mech. Eng. 198, 3534–3550. 10.1016/j.cma.2009.04.015

[B2] Capuano F. Coppola G. Rández L. de Luca L. (2017). Explicit Runge-Kutta schemes for incompressible flow with improved energy-conservation properties. J. Comput. Phys. 328, 86–94. 10.1016/j.jcp.2016.10.040

[B3] Chakraborty P. Balachandar S. Adrian R. J. (2005). On the relationships between local vortex identification schemes. J. Fluid Mech. 535, 189–214. 10.1017/S0022112005004726

[B4] Charnyi S. Heister T. Olshanskii M. A. Rebholz L. G. (2017). On conservation laws of navierðstokes galerkin discretizations. J. Comput. Phys. 337, 289–308. 10.1016/j.jcp.2017.02.039

[B5] Chnafa C. Mendez S. Nicoud F. (2016). Image-based simulations show important flow fluctuations in a normal left ventricle: what could be the implications? Ann. Biomed. Eng. 44, 3346–3358. 10.1007/s10439-016-1614-6 27073110

[B6] Doost S. N. Zhong L. Su B. Morsi Y. S. (2015). The numerical analysis of non-Newtonian blood flow in human patient-specific left ventricle. Comput. Methods Prog. Biomed. 127, 232–247. 10.1016/j.cmpb.2015.12.020 26849955

[B7] Fernández-Pérez G. C. Duarte R. Corral de la Calle M. Calatayud J. Sánchez González J. (2012). Analysis of left ventricular diastolic function using magnetic resonance imaging. Radiología 54, 295–305. 10.1016/j.rxeng.2011.09.003 22226377

[B8] Gao M. Chen C. Zhang S. Qian Z. Vannan M. Rinehart S. (2014). Morphological analysis of the papillary muscles and the trabeculae, in Biomedical Imaging (ISBI), 2014 IEEE 11th International Symposium, (Beijing), 373–376.

[B9] Houzeaux G. Vázquez M. Aubry R. Cela J. M. (2009). A massively parallel fractional step solver for incompressible flows. J. Comput. Phys. 228, 6316–6332. 10.1016/j.jcp.2009.05.019

[B10] Hunt J. C. R. Wray A. A. Moin P. (1988). Eddies, streams, and convergence zones in turbulent flows, in Center for Turbulence Research, Proceedings of the Summer Program, (Stanford, CA), 193–208.

[B11] Imanparast A. Fatouraee N. Sharif F. (2016). The impact of valve simplifications on left ventricular hemodynamics in a three dimensional simulation based on *in vivo* MRI data. J. Biomech. 49, 1482–1489. 10.1016/j.jbiomech.2016.03.021 27040387

[B12] Jofre L. Lehmkuhl O. Ventosa J. Trias F. X. Oliva A. (2013). Conservation properties of unstructured finite-volume mesh schemes for the Navier-Stokes equations. Num. Heat Tran. B Fund. 65, 53–79. 10.1080/10407790.2013.836335

[B13] Khalafvand S. S. Hung T. K. Ng E. Y. K. Zhong L. (2015). Kinematic, dynamic and energy characteristics of diastolic flow in the left ventricle. Comput. Math. Methods Med. 2015:701945. 10.1155/2015/701945 26417381 PMC4568350

[B14] Koullapis P. Kassinos S. C. Muela J. Perez-segarra C. Rigola J. Lehmkuhl O.. (2017). Regional aerosol deposition in the human airways: the SimInhale benchmark case and a critical assessment of *in silico* methods. Eur. J. Pharm. Sci. 113, 77–94. 10.1016/j.ejps.2017.09.003 28890203

[B15] Kulp S. Gao M. Zhang S. Qian Z. Voros S. Metaxas D. (2011). Using high resolution cardiac CT data to model and visualize patient-specific interactions between trabeculae and blood flow. Lect. Notes Comput. Sci. 6891(Pt. 1), 468–475. 10.1007/978-3-642-23623-5 22003651

[B16] Labelle F. Shewchuk J. R. (2007). Isosurface stuffing. ACM Trans. Graph. 26:57 10.1145/1276377.1276448

[B17] Lantz J. Henriksson L. Persson A. Karlsson M. Ebbers T. (2016). Patient-specific simulation of cardiac blood flow from high-resolution CT. J. Biomech. Eng. 138, 1–9. 10.1115/1.4034652 27618494

[B18] Lehmkuhl O. Houzeaux G. Avila M. Owen H. Vazquez M. Mira D. (2017). A low dissipation finite element scheme for the large eddy simulation on complex geometries, in 19h International Conference on Finite Elements in Flow Problems - FEF 2017, (Rome).

[B19] Qi C. (2014). Maximum entropy for image segmentation based on an adaptive particle swarm optimization. Appl. Math. Inform. Sci. 8, 3129–3135. 10.12785/amis/080654

[B20] Standard S. (1999). Characteristics coefficients of soils, in Association of Swiss Road and Traffic Engineers, (Zurich).

[B21] Taylor C. A. Fonte T. A. Min J. K. (2013). Computational fluid dynamics applied to cardiac computed tomography for noninvasive quantification of fractional flow reserve: scientific basis. J. Am. College Cardiol. 61, 2233–2241. 10.1016/j.jacc.2012.11.083 23562923

[B22] Töger J. Kanski M. Carlsson M. Kovács S. J. Söderlind G. Arheden H.. (2012). Vortex ring formation in the left ventricle of the heart: analysis by 4D flow MRI and lagrangian coherent structures. Ann. Biomed. Eng. 40, 2652–2662. 10.1007/s10439-012-0615-3 22805980

[B23] Trayanova N. A. (2011). Whole-heart modeling: applications to cardiac electrophysiology and electromechanics. Circul. Res. 108, 113–128. 10.1161/CIRCRESAHA.110.223610 21212393 PMC3031963

[B24] Trias F. X. Lehmkuhl O. (2011). A self-adaptive strategy for the time integration of navier-stokes equations. Numer. Heat Transf. B 60, 116–134. 10.1080/10407790.2011.594398

[B25] Trias F. X. Lehmkuhl O. Oliva A. Pérez-Segarra C. D. Verstappen R. W. C. P. (2014). Symmetry preserving discretisation of Navier–Stokes equations on collocated unstructured grids. J. Comput. Phys. 258, 246–267. 10.1016/j.jcp.2013.10.031

[B26] Vazquez M. Houzeaux G. Koric S. Artigues A. Aguado-Sierra J. Arís R. (2016). Alya: multiphysics engineering simulation toward exascale. J. Comput. Sci. 14(Suppl. C):15–27. 10.1016/j.jocs.2015.12.007

[B27] Vedula V. Seo J. H. Lardo A. C. Mittal R. (2016). Effect of trabeculae and papillary muscles on the hemodynamics of the left ventricle. Theor. Comput. Fluid Dyn. 30, 3–21. 10.1007/s00162-015-0349-6

[B28] Verstappen R. W. C. P. and Veldman, A. E. P. (2003). Symmetry-preserving discretization of turbulent flow. J. Comput. Phys. 187, 343–368. 10.1016/S0021-9991(03)00126-8

