# Supplementary material for: Left Ventricular Trabeculations Decrease the Wall Shear Stress and Increase the Intra-Ventricular Pressure Drop in CFD Simulations
Source: Front Physiol. 2018 Apr 30;9:458. doi: 10.3389/fphys.2018.00458 (PMC5936785; doi:10.3389/fphys.2018.00458)
Supplement: Supplementary file 1 [file Presentation_1.pdf]

Supplementary Material: Left ventricular trabeculae decrease the wall shear stress and increase the intra-ventricular pressure drop in CFD simulations

Federica Sacco *, **Bruno Paun, Oriol Lehmkuhl, Tinen L. Iles, Paul A. Iaizzo, Guillaume Houzeaux, Mariano Vázquez, Constantine Butakoff and Jazmin Aguado-Sierra** *

*Correspondence:
Federica Sacco:
federica.sacco@bsc.es

Jazmin Aguado-Sierra:
jazmin.aguado@bsc.es

1 SUPPLEMENTARY TABLES

Table 1. Heart database: human characteristics and medical histories

Code	Heart	Sex	Age	BMI[1]	Weight [kg]	Height [m]	Cardiac Medical History	Systemic Medical History
HH_0111	A	M	25	22.2	64.4	1.70	None known	Smoking, substance abuse
HH_0132	B	M	39	25.1	83.9	1.83	CAD[2], hypertension, stent in LAD[3]	Type I diabetes mellitus
HH_0121	C	F	54	21.7	59	1.65	Proximal RCA[4] stenosis	Smoking, thyroid cancer
HH_0141	D	F	24	31.2	86.9	1.67	None known	None known
HH_0084	E	M	36	33.5	103	1.75	Moderate LVH[5], LAE[6] dilated IVC[7], hypertension, cardiac arrhythmia	Smoking, alcoholism

[1] BMI: Body Mass Index = Weight/(Height)^2 [2] CAD: Coronary Artery Disease [3] LAD: Left Anterior Descending Coronary Artery [4] RCA: Right Coronary Artery [5] LVH: Left Ventricular Hypertrophy
[6] LAE: Left Atrial Enlargement [7] IVC: Inferior Vena Cava

Table 2. Total mesh volumes and volume differences between the detailed and smoothed model of each LV

LV	Detailed [m^3]	Smoothed [m^3]	Vol. difference [%]
A	$179 \cdot 10^{-6}$	$175.5 \cdot 10^{-6}$	1.95
B	$209.54 \cdot 10^{-6}$	$182.89 \cdot 10^{-6}$	14.5
C	$175 \cdot 10^{-6}$	$174 \cdot 10^{-6}$	0.57
D	$189.6 \cdot 10^{-6}$	$186 \cdot 10^{-6}$	1.88
E	$236.56 \cdot 10^{-6}$	$265.72 \cdot 10^{-6}$	-12.3

2 SUPPLEMENTARY FIGURES

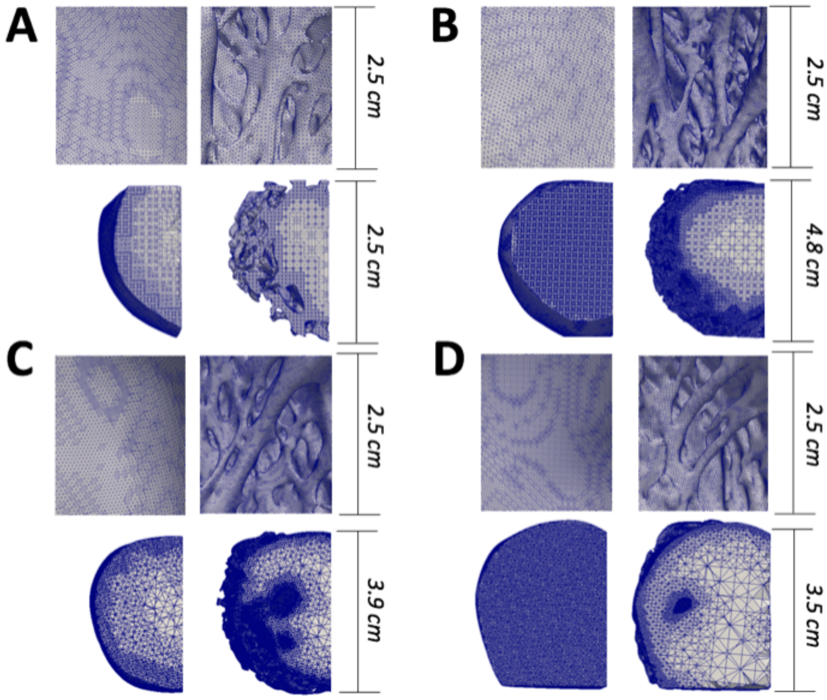

Figure S1. A-D LV myocardial surfaces: smoothed (left) and detailed (right) tetrahedral wire-frame meshes. Shown are inner trabeculations regions (top) and transverse slices (bottom) for each smoothed/detailed pair. Zoomed region actual sizes are reported.

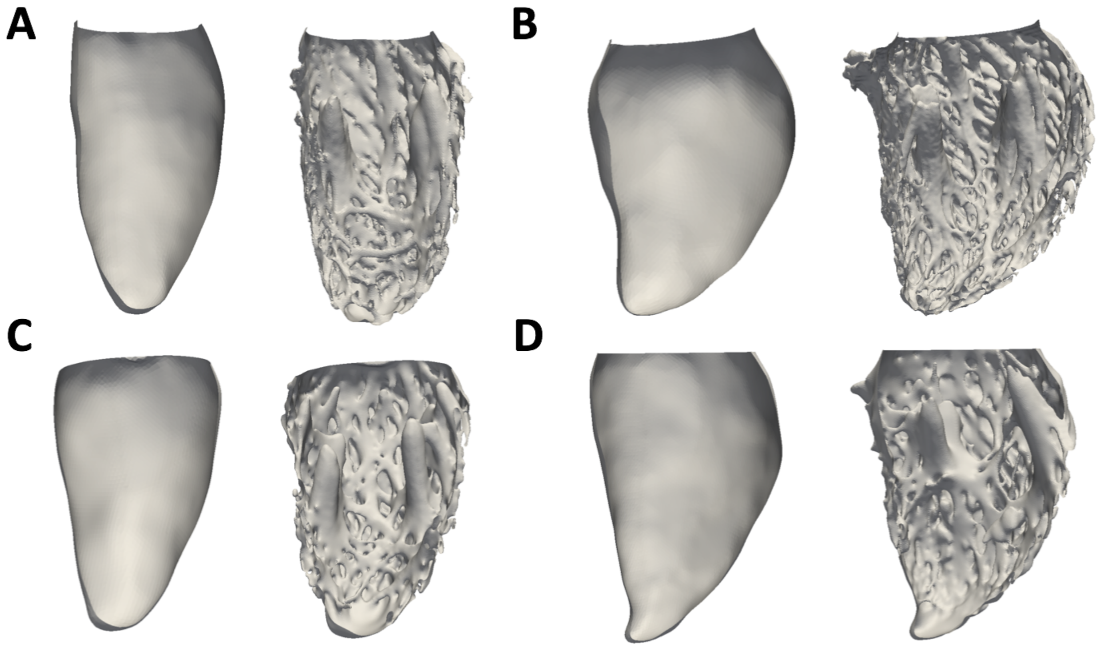

Figure S2. A-D LV sagittal sections: smoothed (left) and detailed (right) endocardial surfaces. PMs and trabeculae are visible inside the detailed LV cavities.

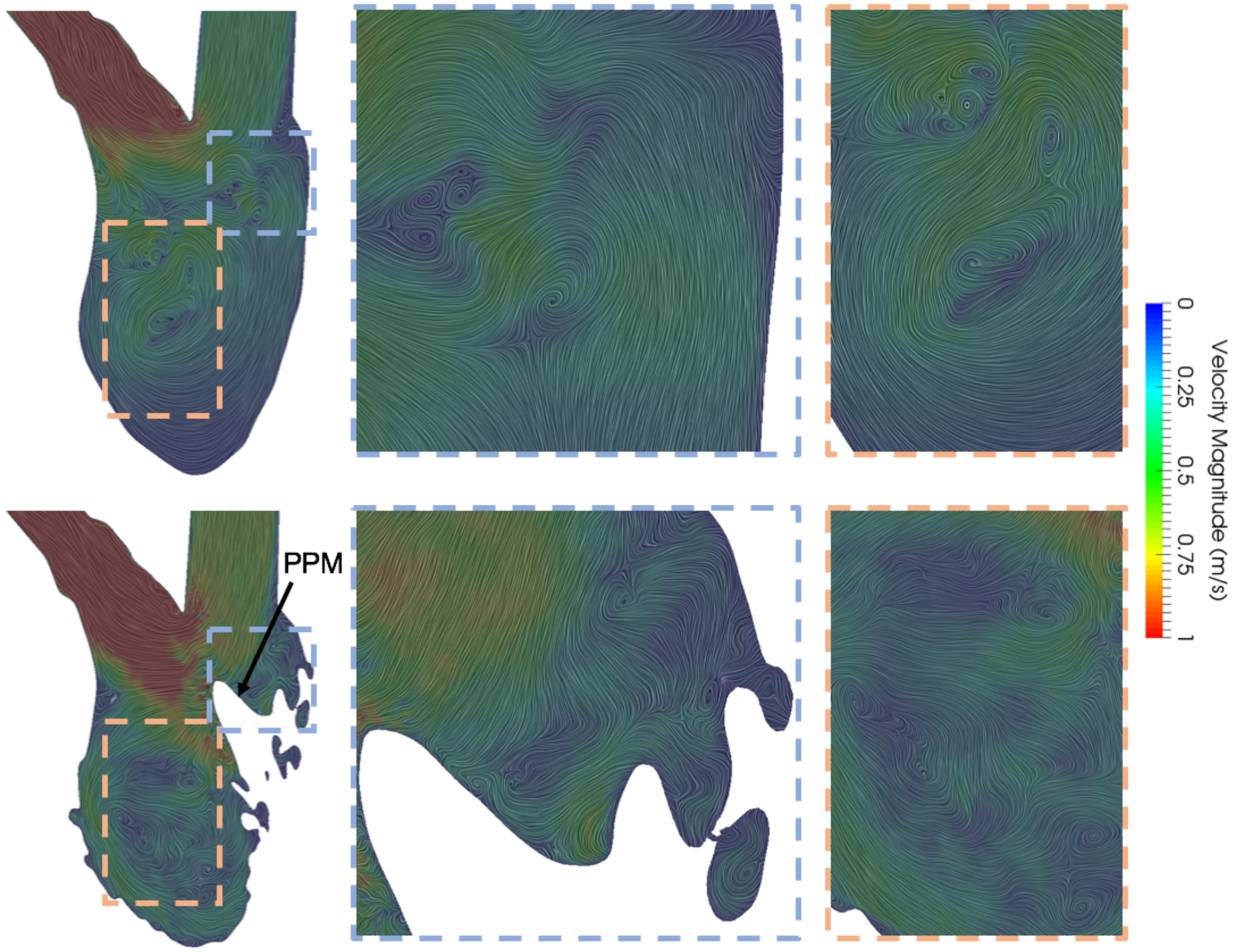

Figure S3. Line integral convolution of smoothed and detailed LV D in constant inflow simulations. In particular, the area right above the posterior PM (PPM) and the center of the ventricular cavity are shown. Blood flow in the detailed LV is more chaotic: the presence of big PMs right below the inlet disturbs the flow markedly generating small scale vortices, absent in the smoothed geometry.
